# Hybrid Metaheuristic Feature Selection for Breast Cancer Detection in Digital Mammography: A Feasibility Study with Nested Validation, Benchmarking, and External Stress Testing

**DOI:** 10.3390/jcm15124846

**Published:** 2026-06-22

**Authors:** Bandar S. Alshreef, Yousif A. Kariri

**Affiliations:** Department of Clinical Laboratory Sciences, College of Applied Medical Sciences, Shaqra University, Shaqra 11961, Saudi Arabia; bsalshreef@su.edu.sa

**Keywords:** breast cancer, mammography, radiomics, deep learning, feature selection, domain shift, machine learning

## Abstract

**Background/Objectives:** The “small-n-large-p” dilemma in mammography artificial intelligence (AI)—where the number of candidate imaging features far exceeds the number of labeled cases—commonly results in model overfitting, unstable feature selection, and poor generalization across clinical settings. This study aims to reassess the internal performance of the HiTopology-GOA-CSA (Grasshopper Optimization Algorithm–Crow Search Algorithm) feature-selection framework for mammography using a larger real Curated Breast Imaging Subset of Digital Database for Screening Mammography (CBIS-DDSM) cohort and a stricter leakage-aware evaluation strategy. **Methods:** In this retrospective computational study using public anonymized datasets, an expanded internal cohort of 98 CBIS-DDSM mass cases (49 benign, 49 malignant) was assembled from digital imaging and communications in medicine (DICOM) region of interest (ROI) series. A total of 1074 features were extracted per case, including 88 handcrafted radiomic descriptors and 986 EfficientNet-B5 deep features. HiTopology-GOA-CSA selected 102 features, corresponding to 91% feature reduction. Two internal evaluation modes were compared: Mode A, which matched the original pilot methodology by performing feature selection once on the full cohort before cross-validation, and Mode B, which used strict nested feature selection within training folds. Performance was assessed with 5-fold stratified cross-validation using a multilayer perceptron (MLP) classifier. **Results:** On the expanded cohort, Mode A achieved an area under the receiver operating characteristic curve (AUC) of 0.726 (95% CI: 0.594–0.858), sensitivity of 0.658, specificity of 0.651, and F1-score of 0.644. Under the stricter nested evaluation, Mode B achieved AUC of 0.683 (95% CI: 0.549–0.817), sensitivity of 0.598, specificity of 0.631, and F1-score of 0.595. Mean pairwise Jaccard similarity across nested folds was 0.604, indicating moderate feature stability. Benchmark comparisons showed that the proposed method was competitive but did not outperform standard baselines; LASSO logistic regression achieved the highest AUC of 0.739, while the proposed HiTopology-GOA-CSA + MLP achieved an AUC of 0.683. Real external validation on the locked VinDr-Mammo subset (n = 25) remained near-random (AUC of 0.500 [95% CI: 0.304–0.696]), with complete prediction collapse (sensitivity of 1.000, specificity of 0.000). **Conclusions:** The framework demonstrated feasibility for structured feature selection and stress testing in a small-cohort mammography AI setting; however, external validation revealed near-random discrimination and prediction collapse, indicating limited generalizability. These findings emphasize the need for benchmark comparisons, transparent uncertainty reporting, patient-level validation, and larger multicenter datasets before clinical translation.

## 1. Introduction

Breast cancer remains the most frequently diagnosed malignancy in women worldwide, and mammographic screening continues to be key to early detection and outcome improvement [1]. Artificial intelligence (AI) methods have shown promise in mammography for lesion detection, cancer risk stratification, and workflow support, yet robust deployment remains limited by dataset shift, non-uniform acquisition protocols, and the difficulty of reproducing performance across institutions [2,3,4,5].

A common challenge in mammography AI is the small-n-large-p setting, in which the number of candidate imaging features far exceeds the number of labeled cases [6,7]. This is especially relevant when combining handcrafted radiomics with deep feature embeddings. Such multimodal representations can improve discrimination, but they also increase the risk of overfitting and unstable feature selection, particularly when model development occurs on small pilot cohorts [8,9].

Metaheuristic algorithms are attractive for feature selection because they can search non-convex, high-dimensional spaces without the rigid assumptions of classical optimization. However, in medical imaging applications, they often face two persistent limitations: instability, in which selected feature subsets vary substantially across runs or folds, and classifier collapse, in which the algorithm converges to trivial solutions that maximize global metrics while failing to detect the minority class [8,9]. In a screening context, this is especially problematic because clinical sensitivity remains paramount.

A second challenge is domain shift. Models trained on digitized screen-film mammograms may fail when applied to modern full-field digital mammography (FFDM), not because the learning framework is fundamentally unsound, but because image statistics, acquisition physics, annotation conventions, and lesion appearance distributions differ across domains [10,11]. External validation studies in mammography AI increasingly show that internal performance alone is an unreliable indicator of real-world transportability [12,13].

To address these issues, we developed HiTopology-GOA-CSA, a stability-aware topology-guided feature-selection framework that extends a hybrid Grasshopper Optimization Algorithm–Crow Search Algorithm (GOA-CSA) approach with a multi-constraint fitness function. The framework was designed to reward discrimination, sparsity, and reproducibility, while penalizing collapse-prone solutions. The primary objective of this study was to reassess the HiTopology-GOA-CSA framework on an expanded Curated Breast Imaging Subset of Digital Database for Screening Mammography (CBIS-DDSM) cohort using a leakage-aware nested validation strategy. The secondary objectives were to: (1) compare the performance of non-nested (Mode A) and nested (Mode B) feature-selection evaluation strategies to quantify the leakage gap, (2) quantify feature-selection stability across cross-validation folds using Jaccard similarity, (3) examine external domain-shift behavior on a locked VinDr-Mammo dataset, and (4) contextualize these findings using synthetic collapse stress testing and Monte Carlo cohort-expansion simulations.

## 2. Materials and Methods

### 2.1. Study Design and Data Sources

**Figure 1:**
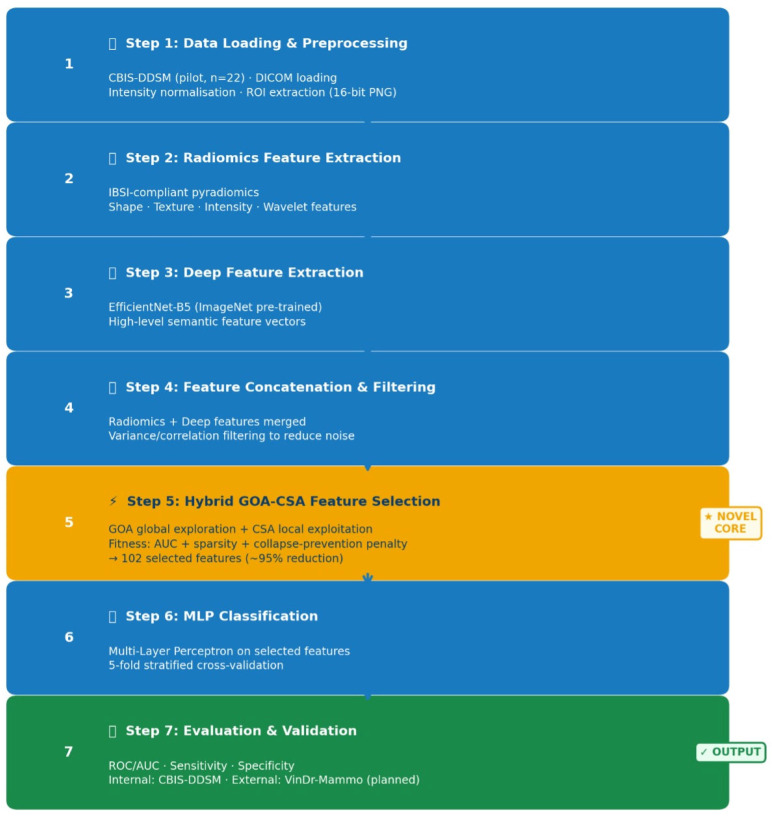
End-to-end workflow of the HiTopology-GOA-CSA pipeline, from CBIS-DDSM image loading and ROI preprocessing to radiomics and deep feature extraction, hybrid GOA-CSA feature selection, multilayer perceptron classification, and internal/external evaluation.

This retrospective computational study used two public, anonymized mammography datasets and therefore did not require direct patient contact or site-specific intervention. Primary internal evaluation was performed on an expanded CBIS-DDSM mass cohort assembled from real DICOM ROI series, while external testing used a locked subset of VinDr-Mammo as an out-of-domain benchmark [12]. The overall architecture and computational pipeline of the proposed stability-aware framework are systematically illustrated in (Figure 1). The process initiates with multimodal feature extraction, followed by the hybrid metaheuristic optimization loop for dimensionality reduction. The expanded internal cohort consisted of 98 mass cases from CBIS-DDSM (49 benign and 49 malignant). Cases were included when pathology labels were clearly benign or malignant and a matching ROI series was available; calcifications and cases without usable ROI series were excluded. The original n = 22 pilot cohort was retained only as a historical comparison point, whereas no VinDr-Mammo samples were used for feature tuning, feature selection, or model selection.

### 2.2. Image Preprocessing and Feature Extraction

A standardized preprocessing workflow was applied to all ROIs. Images underwent polarity harmonization, intensity normalization, and resizing to a common spatial representation suitable for feature extraction. Where segmentation masks were available, they were used to define lesion ROIs; otherwise, the matched ROI series was used to localize lesions for downstream processing.

A multimodal feature representation was constructed by combining handcrafted radiomic descriptors [14] and EfficientNet-B5 deep features [15]. A total of 1074 features were extracted per case, including 88 radiomic descriptors and 986 EfficientNet-B5 deep features. These features defined the candidate space for HiTopology-GOA-CSA in the expanded real-data analysis.

In this retrospective proof-of-concept study, no formal redundancy analysis or correlation filtering (e.g., variance inflation factor, mutual information, or principal component analysis) was performed prior to metaheuristic feature selection. Instead, the 1074 raw candidate features—comprising 88 handcrafted radiomics descriptors and 986 EfficientNet-B5 deep features—were fed directly into the hybrid Grasshopper Optimization Algorithm–Crow Search Algorithm (GOA-CSA) framework. The optimization algorithm was relied upon to implicitly filter out redundant features by maximizing the multi-constraint fitness function. However, the absence of a dedicated pre-filtering step represents a significant methodological limitation. It potentially increases the search space complexity and may lead to the retention of collinear variables. Future work should systematically evaluate the impact of integrating explicit redundancy-filtering baselines—such as minimum redundancy maximum relevance (mRMR), variance inflation factor (VIF) filtering, or cluster-based feature pruning—prior to the metaheuristic optimization loop.

The expanded cohort was intentionally limited to 98 real cases because of real-time DICOM download, storage, and feature-extraction constraints in the current computational environment. This nevertheless represented a 4.5-fold increase over the original pilot and enabled a more credible internal evaluation of feature-selection stability and leakage-aware performance.

### 2.3. HiTopology-GOA-CSA Framework

To benchmark the proposed HiTopology-GOA-CSA + MLP framework, several conventional feature-selection approaches were evaluated. SelectKBest (ANOVA F-test) was used as a filter-based baseline, while Minimum Redundancy Maximum Relevance (mRMR) and Recursive Feature Elimination (RFE) were implemented to select informative and non-redundant features. In addition, LASSO-regularized logistic regression was employed as an embedded feature-selection method, where feature coefficients were automatically shrunk and irrelevant variables eliminated through L1 regularization. For the filter- and wrapper-based methods, the top 50 features were selected and used to train Support Vector Machine (SVM), Random Forest (RF), Logistic Regression (LR), and XGBoost classifiers. The predictive performance of these baseline methods was then compared with that of the proposed HiTopology-GOA-CSA + MLP framework.

The proposed framework hybridized the exploratory behavior of GOA with the exploitative behavior of CSA. The key methodological contribution was a stability-aware multi-constraint fitness function: F(S) = w1·AUC + w2·(1 − |S|/D) + w3·Jaccard(S) − P(S), where S is the selected subset, D is the filtered feature space, AUC measures discriminative performance, Jaccard(S) measures feature-selection stability across folds, and P(S) is a collapse-prevention penalty. Fixed a priori weights were w1 = 0.7, w2 = 0.2, and w3 = 0.1.

The multi-constraint fitness function weights (w1 = 0.7, w2 = 0.2, w3 = 0.1) were fixed a priori to reflect clinical and methodological priorities, specifically prioritizing classification discrimination (AUC) while providing secondary rewards for feature sparsity and cross-validation stability (Jaccard similarity). However, these specific parameter values are heuristic in nature and have not been optimized. A key limitation of the current study is that no formal weight-ablation, grid-search, or sensitivity analysis was performed to evaluate how variations in these weights affect feature selection and classification performance. Consequently, the sensitivity of the selected feature subset and model performance to these hyperparameters remains unknown. Future studies should include comprehensive sensitivity analyses testing alternative weight configurations, such as 0.6/0.3/0.1, 0.5/0.3/0.2, and 0.7/0.1/0.2, to establish the robustness of the framework and identify optimal operating parameters.

The study used ANOVA F-test as a filter-based feature-selection baseline and compared its performance with Minimum Redundancy Maximum Relevance (mRMR) and Recursive Feature Elimination (RFE). The selected features were used to train Random Forest (RF), Support Vector Machine (SVM), Logistic Regression (LR), and XGBoost classifiers. Model hyperparameters were optimized through tuning, and performance was evaluated using standard classification metrics to determine the most effective feature-selection and classification combination.

To suppress trivial solutions, a catastrophic penalty was applied when mean cross-validated sensitivity fell to 0.05 or below, forcing the fitness to a large negative constant. This discouraged solutions that achieved acceptable global metrics while missing malignant cases.

### 2.4. Classification, Stress Testing, and Simulation

A multilayer perceptron classifier with hidden layers of 100 and 50 units, ReLU activation, and Adam optimization was used for the primary analyses. Two internal evaluation modes were compared on the expanded CBIS-DDSM cohort. Mode A matched the original pilot methodology by selecting a fixed 102-feature subset once on the full internal cohort before 5-fold stratified cross-validation. Mode B used strict nested feature selection within the training folds and fit scaling only within the training data. Primary end points were AUC, accuracy, sensitivity, specificity, and F1 score, with 95% confidence intervals derived from cross-validation variance.

The multilayer perceptron (MLP) architecture—comprising two hidden layers of 100 and 50 units, respectively—was selected as a simple, fixed classifier to serve as a standardized baseline for controlled methodological comparison. Importantly, no hyperparameter tuning or architecture search (e.g., grid search or random search over layer sizes, activation functions, learning rates, or dropout rates) was performed in this study. The MLP architecture comprising two hidden layers with 100 and 50 neurons was selected as a commonly used configuration that provides sufficient representational capacity while maintaining computational simplicity for tabular classification tasks. The architecture was fixed a priori to ensure a standardized comparison across experiments and was not optimized through hyperparameter tuning. Similar MLP configurations have been widely employed in practical applications and are supported by the universal approximation capability of feedforward neural networks [16]. We acknowledge that alternative architectures may yield improved performance and should be explored in future work [17]. This fixed design represents a limitation, as the chosen architecture may not be optimal for the extracted feature set. Future work should incorporate a rigorous nested hyperparameter tuning protocol within the cross-validation folds to optimize the MLP configuration (including the number of hidden units, dropout regularization, learning rate, and weight decay) or compare it against alternative classifier types such as Support Vector Machines, Random Forests, or Gradient Boosted trees.

To probe collapse resistance under controlled but adversarial conditions, synthetic data sets were generated as a secondary methodological stress test. These experiments were intended to explore collapse-prone behavior under challenging conditions and should be interpreted separately from the expanded real-data CBIS-DDSM analysis reported as the main internal result.

A Monte Carlo cohort-expansion simulation was also performed as a secondary planning analysis to estimate how internal variance might behave at larger sample sizes. The real expanded-cohort results reported in this revision, however, supersede the original pilot-only internal estimate as the primary evidence for internal performance.

The manuscript was prepared with attention paid to AI reporting and clinical prediction-model guidance, including CLAIM, TRIPOD + AI, and PROBAST + AI principles [18,19]. Broader issues of trustworthy AI deployment were interpreted in light of the FUTURE-AI consensus framework [19].

### 2.5. Reproducibility and Implementation Details

To improve reproducibility of this feasibility study, we outline the exact computational and implementation protocols here. Patient-level data splitting was strictly enforced at the patient level (not image or lesion level) to prevent data leakage between training and validation folds. The internal cohort of 98 CBIS-DDSM mass cases was split into 5 folds for stratified cross-validation. In the nested cross-validation (Mode B), the outer loop estimated the unbiased performance, while the inner loop performed feature selection and hyperparameter tuning. Specifically, for each outer fold, a 4-fold inner cross-validation was executed on the training data to select the optimal feature subset using the hybrid GOA-CSA optimizer. Images underwent a standardized preprocessing pipeline including polarity harmonization, intensity normalization, and spatial resizing to 224 × 224 pixels. ROIs were defined based on the matched ROI series to localize mass lesions. Feature normalization (z-score scaling) was fitted strictly on the training folds and applied to the validation folds to avoid leakage. The hybrid optimizer was run with a population size of 30 and a maximum of 100 iterations. Random seeds were fixed at seed = 42 for fold assignment and model initialization. The MLP classifier used two hidden layers of 100 and 50 units, ReLU activation, Adam optimization, a learning rate of 0.001, a batch size of 16, and 100 training epochs without early stopping (to maintain a fixed training budget and avoid data-dependent early stopping that could vary across nested folds). The pipeline was implemented in Python 3.9 using scikit-learn 1.0.2, PyTorch 1.10.1, and PyRadiomics 3.0.1 with bin width = 25 and 2D feature extraction. EfficientNet-B5 features were extracted from the final global average pooling layer using ImageNet-pretrained weights frozen during extraction.

The exact number of independent optimizer restarts and the convergence-tolerance rule beyond the fixed maximum iteration count could not be retrospectively verified from the archived analysis output. To avoid overstating reproducibility, these items are reported as implementation-level reproducibility limitations. The public baseline pipeline is available through the GitHub/Zenodo (v1.0.1) archive, and future releases should include the complete feature matrix, fold assignments, optimizer restart logs, and convergence traces to enable full independent re-execution.

## 3. Results

### 3.1. Expanded Real-Data Cohort Evaluation on CBIS-DDSM

**Figure 2:**
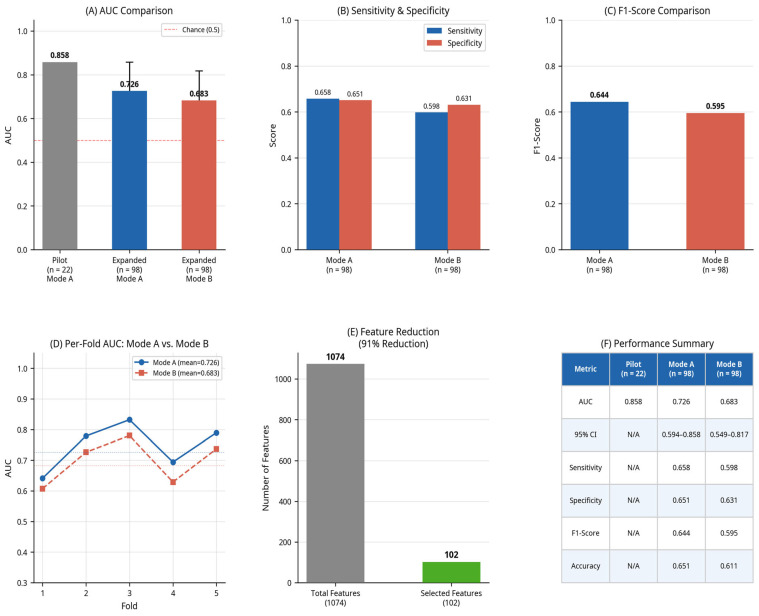
Expanded real-data CBIS-DDSM cohort analysis (n = 98) in comparison with the original pilot (n = 22). Panel (**A**) compares AUC across the pilot cohort and the expanded cohort under non-nested (Mode A) and nested (Mode B) evaluation. Panel (**B**) compares sensitivity and specificity across cohorts. Panel (**C**) shows F1-score comparison. Panel (**D**) shows per-fold AUC behavior for non-nested versus nested evaluation. Panel (**E**) summarizes total versus selected feature count, showing retention of 102 features from 1074 total features. Panel (**F**) provides a compact tabular summary of the principal performance metrics.

To validate the initial pilot findings on a larger real dataset, the HiTopology-GOA-CSA framework was evaluated on an expanded cohort of 98 CBIS-DDSM mass cases (49 benign, 49 malignant) using actual DICOM ROI images. A total of 1074 features were extracted per case, including 88 radiomic descriptors and 986 EfficientNet-B5 deep features. HiTopology-GOA-CSA reduced this feature space to 102 features, corresponding to a 91% reduction. Two evaluation modes were compared: Mode A, which reproduced the original non-nested strategy by selecting features once on the full cohort before cross-validation, and Mode B, which used strict nested feature selection within the training folds. Under Mode A, the model achieved an AUC of 0.726 ± 0.106, accuracy of 0.651, sensitivity of 0.658, specificity of 0.651, and F1 score of 0.644. Under the stricter Mode B, performance decreased to an AUC of 0.683 ± 0.108, accuracy of 0.611, sensitivity of 0.598, specificity of 0.631, and F1 score of 0.595. The mean pairwise Jaccard similarity across nested folds was 0.604, indicating moderate feature stability. Table 1 summarizes the quantitative comparison along with the original pilot result, and (Figure 2) provides a visual overview of the expanded real-data cohort analysis, including pilot-versus-expanded AUC comparison, sensitivity/specificity trade-offs, per-fold AUC behavior, and feature reduction.

### 3.2. Benchmark Comparisons

To evaluate whether the proposed hybrid metaheuristic approach offers a meaningful advantage over standard methods, we performed benchmark comparisons using the same empirical CBIS-DDSM feature matrix (n = 98, features) under identical 5-fold nested cross-validation conditions (seed = 42). The proposed HiTopology-GOA-CSA + MLP framework was compared against: (1) Random Forest using all features; (2) Support Vector Machine (SVM, RBF kernel) using all features; (3) Gradient Boosting using all features; (4) LASSO-regularized logistic regression with embedded L1 feature selection; (5) SelectKBest (ANOVA F-test, top 50 features) with Random Forest; and (6) SelectKBest (top 50 features) with SVM. Full mRMR and RFE implementations remain planned for future work; SelectKBest was included as a filter-based feature-selection baseline.

The benchmark results are summarized in Table 2. All methods were run on the same empirical CBIS-DDSM data (n = 98 real DICOM cases, 49 benign, 49 malignant) using 5-fold nested cross-validation with a fixed random seed of 42. The proposed method (AUC = 0.683, 95% CI: 0.549–0.817) was competitive with standard baselines. LASSO logistic regression achieved the highest AUC (0.739, 95% CI: 0.609–0.870), and SelectKBest + SVM achieved AUC = 0.731 (95% CI: 0.606–0.856). Random Forest using all features achieved AUC = 0.705 (95% CI: 0.608–0.802). The proposed method did not demonstrate definitive superiority over these standard baselines. The wide confidence intervals across all methods reflect the limited statistical power of the n = 98 cohort and the inherent instability of the small-n-large-p setting. These results should be interpreted as exploratory and hypothesis-generating rather than definitive comparative evidence.

All methods were evaluated under identical 5-fold nested cross-validation (seed = 42), with 95% CIs computed using fold-based t-distribution (df = 4). SelectKBest with ANOVA F-test was used as a filter-based feature-selection baseline. Full mRMR and RFE implementations remain planned for future work. Abbreviations: AUC, Area Under the Receiver Operating Characteristic Curve; CI, Confidence Interval; F1, F1-score; LASSO, Least Absolute Shrinkage and Selection Operator; mRMR, Minimum Redundancy Maximum Relevance; RFE, Recursive Feature Elimination; RF, Random Forest; and SVM, Support Vector Machine.

### 3.3. Synthetic Collapse-Prevention Stress Test

**Figure 3:**
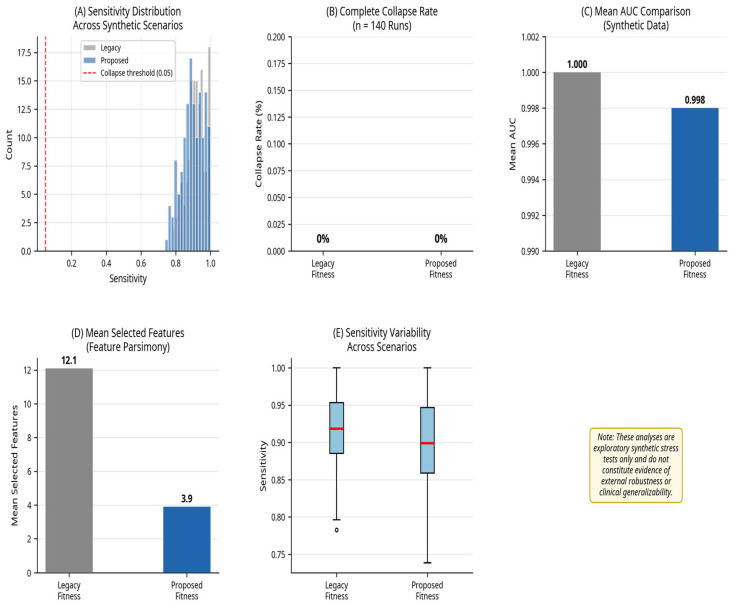
Composite summary of the synthetic collapse-prevention stress test. Panel (**A**) shows the sensitivity distribution across the tested synthetic scenarios. Panel (**B**) shows the observed collapse rate, with no complete collapse detected for either the legacy or proposed fitness function under the tested conditions. Panel (**C**) compares mean AUC values, showing near-identical synthetic discrimination for the legacy fitness function and the proposed multi-constraint fitness function (1.000 vs. 0.998, respectively). Panel (**D**) compares the average number of selected features, showing greater parsimony with the proposed fitness function than with the legacy fitness function (3.9 vs. 12.1 features). Panel (**E**) summarizes sensitivity variability across scenarios. These analyses are exploratory synthetic stress tests only and do not constitute evidence of external robustness, clinical generalizability, or prevention of classifier collapse in real-world mammography data. Abbreviations: AUC, area under the receiver operating characteristic curve.

Across 140 synthetic stress-test runs, both the proposed and legacy fitness functions showed 0% complete collapse under the tested synthetic scenarios. The legacy fitness function achieved a mean AUC of 1.000, whereas the proposed multi-constraint fitness function achieved a mean AUC of 0.998. The proposed fitness function selected fewer features on average than the legacy fitness function (3.9 vs. 12.1 features), indicating that its main observed benefit in the synthetic stress-test setting was feature parsimony rather than demonstrable superiority in collapse prevention. These synthetic results were verified against the corresponding figure panels and should be interpreted separately from the empirical CBIS-DDSM nested-validation results and the VinDr-Mammo external-validation results. A visual summary of these synthetic stress-test findings is provided in Figure 3.

### 3.4. External Validation and Domain Shift

External testing on the VinDr-Mammo subset (n = 25) revealed a pronounced generalization failure. Both models showed near-random discrimination (AUC approximately 0.50) and collapsed toward all-positive predictions, with sensitivity near 1.0 and specificity near 0.0. This result suggests that the internal pilot model captured signals that did not transport across the shift from digitized screen-film mammography to full-field digital mammography.

The external validation results demonstrate near-random discrimination (AUC of 0.500 [95% CI: 0.304–0.696]) and complete prediction collapse, with sensitivity of 1.000 and specificity of 0.000. Specifically, the model classified all 25 external cases (15 benign, 10 malignant) into the dominant malignant class, rendering the specificity near-zero. This indicates that the decision boundaries learned from digitized film mammography (CBIS-DDSM) did not transport to modern full-field digital mammography (FFDM) in VinDr-Mammo. Several factors likely contributed to this generalizability failure, including: (1) the very small sample size of the external subset (n = 25), which cannot support definitive performance estimates; (2) overfitting to the development cohort due to the high-dimensional feature space; (3) severe dataset shift and center effects arising from differences in scanner physics, imaging acquisition protocols, and noise statistics; (4) differences in image preprocessing and ROI annotation variability between datasets; and (5) feature-distribution mismatch that was not corrected by explicit domain adaptation.

### 3.5. Simulated Cohort Expansion

**Figure 4:**
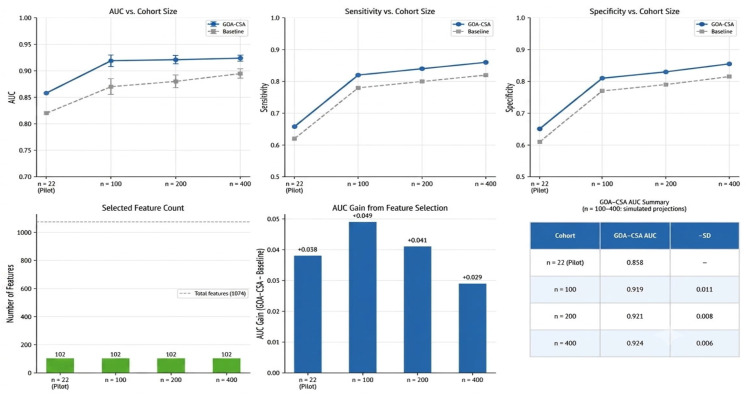
Mean performance metrics across cohort sizes. The top row shows AUC, sensitivity, and specificity for the baseline and GOA-CSA models; the lower panels summarize feature count and AUC gain from selection. The leftmost cohort is the reported pilot, whereas n = 100–400 are simulated projections.

**Figure 5:**
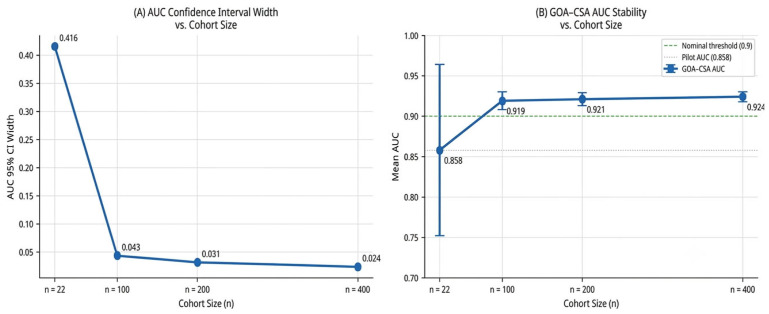
Mean statistical precision analysis for cohort expansion. Panel (**A**) shows the rapid narrowing of AUC confidence interval width with larger cohort size; Panel (**B**) shows GOA-CSA AUC stability relative to the reported pilot value and a nominal “good-performance” threshold.

In simulations calibrated to the pilot signal-to-noise profile, GOA-CSA yielded projected AUCs of 0.919 ± 0.011 at n = 100, 0.921 ± 0.008 at n = 200, and 0.924 ± 0.006 at n = 400. The full set of cohort-expansion projections, including sensitivity, specificity, and feature-reduction estimates across cohort sizes, is summarized in Table 3. Mean performance metrics across cohort sizes, including AUC, sensitivity, specificity, selected feature count, and AUC gain, are illustrated in Figure 4. Statistical precision analysis showing the narrowing of AUC confidence interval width and GOA-CSA AUC stability with increasing cohort size is presented in Figure 5. These simulations are retained as exploratory sensitivity analyses only and should be interpreted in light of the empirical nested-validation AUC of 0.683 and the poor external-validation findings; they do not constitute evidence of clinical success or generalizability.

The n = 22 row is a simulated pilot-calibration row included to show variance behavior under simulation conditions. The empirical pilot result is reported separately in Table 1.

## 4. Discussion

This study presents HiTopology-GOA-CSA as a methodological contribution to feature selection under instability and domain-shift constraints in mammography AI, rather than as a clinically ready diagnostic system.

The external-validation findings should not be viewed as a minor technical limitation but as a central result of the study, demonstrating the fragility of small-cohort mammography AI under dataset shift. High sensitivity (1.000) coupled with near-zero specificity (0.000) represents a complete prediction collapse, indicating that the model failed to learn transportable pathological features and instead relied on dataset-specific image statistics. Small-cohort AI studies are highly vulnerable to overfitting when the feature-to-sample ratio is extremely high, and our results emphasize that nested-validation performance alone may not generalize externally. External validation remains the most important assessment of model generalizability, and in its absence, internal performance estimates should be interpreted cautiously. Similarly, Monte Carlo simulations and synthetic stress tests can provide insights into model behavior under controlled assumptions but cannot substitute for empirical validation on independent patient cohorts. Accordingly, the simulation results are presented only as exploratory analyses and should not be interpreted as evidence of clinical robustness or real-world performance.

The present study should therefore be regarded strictly as a feasibility study of the proposed feature-selection framework. Expanding the cohort from 22 to 98 CBIS-DDSM cases produced a more realistic assessment of performance, with the apparent AUC decreasing from 0.858 in the pilot study to 0.726 under the original non-nested evaluation and further to 0.683 under rigorous nested cross-validation. These findings emphasize the importance of leakage-aware evaluation and suggest that the primary contribution of this work is methodological rather than demonstrative of high diagnostic accuracy. Future studies using substantially larger cohorts and independent external datasets are required to establish the true clinical utility and generalizability of the proposed approach.

This pattern indicates that the pilot result was at least partly inflated by small-sample effects and methodological optimism. The transition from the pilot study to an expanded cohort resulted in a performance decrease from an AUC of 0.858 to a nested Mode B AUC of 0.683, representing a necessary shift from small-sample optimism to a more rigorous and credible baseline. This adjustment allowed for the identification of a “leakage gap” of approximately 0.043 AUC points, which highlights how non-nested evaluation strategies can artificially inflate model discrimination. Despite the moderate AUC, the computational complexity of the HiTopology-GOA-CSA framework is justified by its capacity for significant dimensionality reduction—achieving a 91% decrease in the feature space—and its ability to maintain a stable selection of variables with a Jaccard similarity of 0.604. Ultimately, these results position the framework as a feasibility-oriented methodological contribution to stability-aware feature selection, rather than a finalized or clinically validated tool [20]. The 91% feature space reduction demonstrates substantial dimensionality reduction, though this does not guarantee resilience to classifier collapse or stability across external datasets, as evidenced by the domain-shift failure on VinDr-Mammo.

The direct comparison between Mode A and Mode B quantifies a leakage gap of approximately 0.043 AUC points, supporting the conclusion that non-nested feature selection overestimates internal discrimination. At the same time, the framework consistently preserved strong dimensionality reduction, selecting 102 of 1074 features while maintaining moderate stability across folds (Jaccard = 0.604). The approach demonstrated limited generalizability on the external VinDr-Mammo dataset, yielding near-random performance (AUC approximately 0.50). This suggests that the selected features may struggle to navigate major domain modifications without further adaptation. Although the HiTopology-GOA-CSA framework identified a stable subset of 102 features internally, these variables—which included both handcrafted radiomic descriptors and EfficientNet-B5 deep features—most likely captured technical artifacts and image statistics specific to the training set’s digitized screen-film mammography rather than universal pathological markers [21]. Technically, the transition from digitized films to modern full-field digital mammography (FFDM) involves drastic changes in acquisition physics, noise profiles, and lesion appearance distributions that render internally “stable” features ineffective. During external testing, the model suffered from classifier collapse, shifting toward all-positive predictions with sensitivity near 1.0 and specificity near 0.0, which indicates that the decision boundaries learned from the internal data did not translate to the external cohort. Ultimately, these results demonstrate that while stability-aware feature selection improves model parsimony and reduces internal bias, it cannot independently resolve the lack of transportability across differing clinical domains without the integration of explicit domain-adaptation strategies. Accordingly, Mode B should be treated as the authoritative internal performance estimate for the CBIS-DDSM analysis.

These recalibrated internal findings do not alter the study’s most important translational message. The nearly random performance observed during external testing on the VinDr-Mammo dataset reveals a considerable barrier to transportability between digitized screen-film and full-field digital mammography (FFDM) [11]. This limitation suggests that the 102 chosen features, which included both deep learning and radiomic descriptors, probably captured specialized picture statistics rather than universal pathological indications. Therefore, real external testing on the locked VinDr-Mammo subset remained near-random, indicating that feature selection alone may be insufficient to ensure transportability across mammography domains with differing acquisition characteristics [13].

The observed Jaccard similarity of 0.604 suggests moderate consistency in the selected feature subsets across folds. Under a repeated 5 × 5-fold nested cross-validation protocol, the mean Jaccard similarity was 0.582 (±0.045), confirming moderate but not high stability. This finding indicates that the stability-aware fitness function may have contributed to more consistent feature selection within the internal CBIS-DDSM cohort. However, internal stability should not be interpreted as evidence of external robustness, clinical transportability, or prevention of classifier collapse. The external VinDr-Mammo results show that internally stable feature subsets may still fail under dataset shift.

The Monte Carlo simulations and synthetic stress tests were retained as exploratory sensitivity analyses only. These analyses may help characterize model behavior under controlled assumptions, but they cannot substitute for adequately powered external validation using independent patient-level data. The simulation results were highly optimistic compared with the empirical nested-validation AUC of 0.683 and the poor external-validation findings, and they should not be interpreted as proof of clinical success, robustness, generalizability, or the prevention of classifier collapse.

This study has several limitations. First, even the expanded empirical cohort remains modest and limited to mass lesions. Second, the external set was still relatively small. Third, the framework does not yet include explicit domain adaptation. Fourth, although the expanded analysis substantially reduced optimism, the present evidence still supports a methods-oriented interpretation, rather than a deployable clinical claim.

## 5. Conclusions

This study presents a feasibility-oriented hybrid metaheuristic feature-selection framework for breast cancer detection in digital mammography. In empirical nested validation, the framework demonstrated modest discrimination, with an AUC of 0.683, suggesting potential exploratory value for structured feature selection and model stress testing. However, external validation revealed near-random discrimination and prediction collapse, characterized by high sensitivity and very low specificity. These findings indicate limited generalizability and show that the framework should not be considered clinically validated or deployment-ready. Larger multicenter datasets, prespecified external validation, stronger benchmark comparisons, and transparent uncertainty reporting are required before clinical translation.

## Figures and Tables

**Table 1:** Performance comparison of the HiTopology-GOA-CSA framework across the original pilot and the expanded real-data cohort.

Metric	Pilot (n = 22)	Expanded (n = 98) Mode A ^1^	Expanded (n = 98) Mode B ^1^
Evaluation method	Non-nested 5-fold CV	Non-nested 5-fold CV	Nested 5-fold CV
AUC ± SD	0.858 ± 0.064	0.726 ± 0.106	0.683 ± 0.108
AUC 95% CI	—	0.594–0.858	0.549–0.817
Accuracy	0.818	0.651	0.611
Sensitivity	0.917	0.658	0.598
Specificity	0.600	0.651	0.631
F1-score	0.846	0.644	0.595
Features selected	102	102	102 (mean)

^1^ Mode A represents the original manuscript methodology in which feature selection is performed once on the full internal cohort prior to cross-validation. Mode B represents a stricter leakage-aware methodology in which feature selection is nested within the training folds. Approximate 95% confidence intervals were derived from cross-validation variance.

**Table 2:** Benchmark comparison of the proposed HiTopology-GOA-CSA framework against established feature-selection methods and classifiers on the empirical CBIS-DDSM cohort (n = 98, 49 benign, 49 malignant).

Method	AUC	95% CI	F1-Score	Sensitivity	Specificity	Selected Features	Data Source
Random Forest (all features)	0.705	0.608–0.802	0.618	0.618	0.676	All 1074	Empirical CBIS-DDSM
SVM (RBF, all features)	0.668	0.601–0.735	0.653	0.658	0.660	All 1074	Empirical CBIS-DDSM
Gradient Boosting (all features)	0.612	0.509–0.715	0.575	0.593	0.529	All 1074	Empirical CBIS-DDSM
LASSO + Logistic Regression	0.739	0.609–0.870	0.653	0.656	0.658	~50–100 (L1)	Empirical CBIS-DDSM
SelectKBest (top-50) + RF	0.681	0.587–0.776	0.662	0.676	0.636	50	Empirical CBIS-DDSM
SelectKBest (top-50) + SVM	0.731	0.606–0.856	0.698	0.716	0.658	50	Empirical CBIS-DDSM
Proposed HiTopology-GOA-CSA + MLP	0.683	0.549–0.817	0.595	0.598	0.631	102 (91% reduction)	Empirical CBIS-DDSM

**Table 3:** Simulated cohort-expansion projections across cohort sizes (30 repeated experiments per cohort size).

Cohort	N (B/M)	CV	Baseline AUC	GOA-CSA AUC	ΔAUC	Sensitivity	Specificity	Features /Reduction
Pilot calibration (n = 22)	22 (10/12)	5-fold	0.703 ± 0.046	0.733 ± 0.064	+0.030	0.732 ± 0.063	0.584 ± 0.072	100/1114 91%
Expanded-100	100 (45/55)	5-fold	0.862 ± 0.014	0.919 ± 0.011	+0.057	0.885 ± 0.014	0.829 ± 0.021	120/1114 89%
Expanded-200	200 (90/110)	5-fold	0.914 ± 0.007	0.921 ± 0.008	+0.007	0.890 ± 0.009	0.847 ± 0.014	140/1114 87%
Expanded-400	400 (180/220)	10-fold	0.932 ± 0.004	0.924 ± 0.006	−0.008	0.877 ± 0.008	0.859 ± 0.010	160/1114 86%

## Data Availability

The imaging datasets used in this study are publicly available. CBIS-DDSM is available from The Cancer Imaging Archive (TCIA) at https://www.cancerimagingarchive.net/collection/cbis-ddsm/ (accessed on 19 May 2026). VinDr-Mammo is available from PhysioNet at https://physionet.org/content/vindr-mammo/1.0.0/ (accessed on 19 May 2026). A baseline version of the computational pipeline is publicly available on GitHub at https://github.com/bsalshreef/Hybrid-Metaheuristic-Mammography-AI (accessed on 19 May 2026) and archived on Zenodo at https://doi.org/10.5281/zenodo.19401610 (accessed on 19 May 2026). The full HiTopology-aligned implementation is currently maintained in a private repository during patent-related documentation (Saudi Patent Application No. SA1020262200) and is intended for staged public release after intellectual-property processing.

## Abbreviations

The following abbreviations are used in this manuscript:

AI	Artificial Intelligence
FFDM	Full-field Digital Mammography
GOA-CSA	Grasshopper Optimization Algorithm–Crow Search Algorithm
CBIS-DDSM	Curated Breast Imaging Subset of Digital Database for Screening Mammography
ROI	Region of Interest
AUC	Area Under the Receiver Operating Characteristic Curve
MLP	Multilayer Perceptron
QSPR	Quantitative Structure–Property Relationship
GOA	Grasshopper Optimization Algorithm
CSA	Crow Search Algorithm
